# Favorable response to multimodal treatment in hepatocellular carcinoma with inferior vena cava and right atrial tumor thrombus and left adrenal gland metastasis: A case report and literature review

**DOI:** 10.1097/MD.0000000000027987

**Published:** 2021-12-10

**Authors:** Ning Sun, Jialin Zhang, Baifeng Li, Ailin Li, Mutian Lv, Chengshuo Zhang

**Affiliations:** a Department of Hepatobiliary Surgery, The First Affiliated Hospital of China Medical University, Shenyang, Liaoning, China; b Department of Radiation Oncology, The First Affiliated Hospital of China Medical University, Shenyang, Liaoning, China; c Department of Nuclear Medicine The First Affiliated Hospital of China Medical University, Shenyang, Liaoning, China.

**Keywords:** hepatocellular carcinoma, PD-1 inhibitor, radiotherapy, sorafenib, transcatheter arterial chemoembolization

## Abstract

**Rationale:**

Hepatocellular carcinoma (HCC) is the fourth most common cause of cancer-related deaths and the sixth most commonly diagnosed cancer globally. Interdisciplinary and multimodal treatment strategies are essential for a successful therapy in HCC. Established therapies for HCC treatment include surgical resection, liver transplantation, local ablative therapies, transarterial chemoembolization (TACE), tyrosine kinase inhibitors (TKIs), immunotherapy, and radiotherapy (RT).

**Patient concerns:**

A 52-year-old male patient did an ultrasound scan and found a large mass within the right lobe of the liver and gallstones in December 2018. He had a history of chronic hepatitis C virus infection (30 years) and was treated with sofosbuvir (400 mg, q.d.) for 1 year. The patient never had any symptoms of gallstones. Enhanced abdominal computed tomography of this patient showed a heterogeneous irregular mass with the largest measurement of up to 13.7 × 11.1 cm in size in the right lobe of the liver, meanwhile also had inferior vena cava (IVC) tumor thrombus, right atrial (RA) tumor thrombus, and left adrenal gland metastasis. The laboratory test data revealed that the serum tumor marker α-fetoprotein was 2.63 ng/mL, cancer antigen 19-9 (CA 19-9) was 34.40 U/mL, and protein induced by Vitamin K absence was 391.94 mAU/mL.

**Diagnosis:**

HCC with IVC tumor thrombus, RA tumor thrombus, and left adrenal gland metastasis, and gallstones.

**Interventions:**

He was hospitalized and received TACE treatment, oral TKIs, intravenous drip programmed cell death-1 (PD-1) inhibitor and RT.

**Outcomes:**

The patient showed a favorable response after consecutive treatment with TACE, TKIs, PD-1 inhibitor, and RT. Until now, the patient has survived 34 months since the diagnosis of the disease.

**Lessons:**

Our case suggests that TACE combined with TKIs, PD-1 inhibitor, and RT may be a suitable treatment option for advanced HCC patients with IVC tumor thrombus and/or RA tumor thrombus, and/or adrenal gland metastasis.

## 1 Introduction

Hepatocellular carcinoma (HCC) is the fourth most common cause of cancer-related deaths and the sixth most commonly diagnosed cancer globally.^[1]^ Treatment for HCC is guided by the Barcelona Clinic Liver Cancer (BCLC) system, which recommends certain therapies, depending on the cancer stage.^[2]^ Liver resection and liver transplant yield the most favorable outcomes, but are only recommended in the early stages of liver cancer. HCC with inferior vena cava (IVC) and right atrial (RA) tumor thrombus has a poor prognosis and few treatment options. Surgical approaches, including hepatectomy and removal of the tumor thrombus are currently the most frequently reported treatment modality. However, most patients with advanced HCC are not eligible for complex surgical interventions due to reduced liver function, potential postoperative complications, preoperative tumor metastases, and early recurrence.^[3,4]^ Therefore, there is an urgent need for the development of a systemic and comprehensive treatment for advanced HCC.

Systemic treatment options for HCC, such as transarterial chemoembolization (TACE) and transarterial radioembolization, have expanded in recent years. These treatment modalities are used for HCC confined to the liver or to decrease tumor size prior to surgical intervention. Sorafenib and lenvatinib administration are recommended first line treatments for advanced stage HCC.^[5–7]^ In 2017, the Food and Drug Administration approved a novel programmed cell death 1 (PD-1) checkpoint inhibitor known as nivolumab. This drug exhibited promising survival outcomes in patients with the disease progression or patients experiencing unacceptable adverse effects as result of sorafenib treatment. With the development of radiotherapy (RT) techniques, favorable outcomes in prospective studies as well as the clinical application of liver-directed RT have been consistently reported.^[8–10]^

In this report, we describe the case of an HCC patient with IVC tumor thrombus, RA tumor thrombus, and left adrenal gland metastasis who showed a favorable response after treatment with TACE, tyrosine kinase inhibitors (sorafenib, regorafenib), PD-1 inhibitor (nivolumab and toripalimab), and RT. To the best of our knowledge, this represents is the first reported case of HCC with the aforementioned features and treatment modalities, and the patient survived the longest.

## 2 Case presentation

A 52-year-old male was referred to our department in December, 2018 due to an ultrasound scan showing gallstones and a large mass within the right lobe of the liver. He had a history of chronic hepatitis C virus infection (30 years) and was treated with sofosbuvir (400 mg, q.d.) for 1 year. The patient never had any symptoms of gallstones.

**Figure 1:**
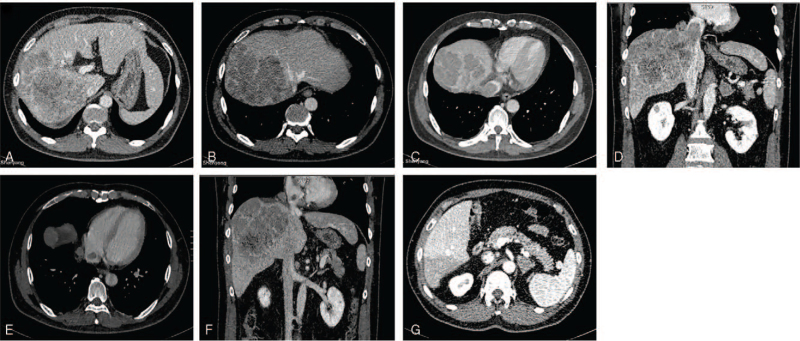
A, B: Enhanced CT revealed a mass tumor in the right lobe of liver, also invaded part of the middle hepatic vein; C, D: The tumor thrombus in the inferior vena cava; E, F: The tumor thrombus in the right atrial; G: Left adrenal gland metastasis. CT = computed tomography.

Enhanced abdominal computed tomography (CT) showed a heterogeneous irregular mass with the largest measurement of up to 13.7 × 11.1 cm in size in the right lobe of the liver. The patient also had IVC tumor thrombus, RA tumor thrombus, and left adrenal gland metastasis (Fig. 1).

**Figure 2:**
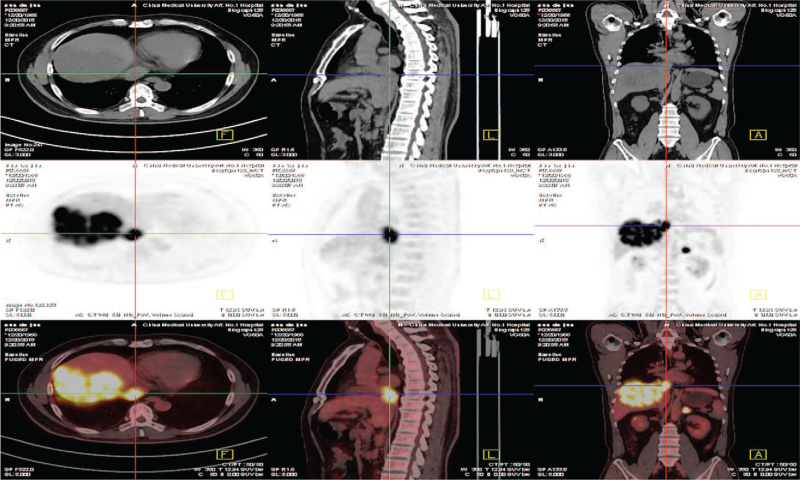
PET-CT showed the high FDG of the mass tumor in the right lobe of liver, the tumor thrombus in the inferior vena cava; the tumor thrombus in the right atrial and left adrenal gland metastasis. FDG = fluorodeoxyglucose, PET-CT = positron emission tomography-CT.

Positron emission tomography-CT showed the fluorodeoxyglucose of the right lobe of the liver, IVC, RA, left adrenal gland, and right inguinal were increased and the largest standardized uptake value values were 23.6, 18.6, 18.3, and 18.5, respectively (Fig. 2).

The laboratory test data revealed that the serum tumor marker α-fetoprotein (AFP) was 2.63 ng/mL, cancer antigen (CA) was 19-9 34.40 U/mL, and protein induced by Vitamin K absence (PIVKA) was 391.94 mAU/mL (Table 1). The hepatitis C virus RNA load was <1.5E1 and the liver function was satisfactory: Total bilirubin was 14.0 μmol/L, direct bilirubin was 5.5 μmol/L, gamma-glutamyl transpeptidase was 153 U/L, alanine aminotransferase (ALT) was 39 U/L, aspartate aminotransferase (AST) was 39 U/L, serum albumin was 43.9 g/L, and prothrombin time was 13.7 second (Table 1). The patient's tumor stage was confirmed with a clinical diagnosis of BCLC stage C, Child-Pugh class A (score 5), and an Eastern Cooperative Oncology Group Performance Score of 1.

**Table 1:** The dynamic change of the liver function, PT, thyroid function, and tumor markers.

	Reference range	Before treatment	After treatment			
Follow-up		2018-12	2019-4	2020-4	2020-11	2021-7
Liver function						
AST (U/L)	13-35	39	35	30	31	19
ALT (U/L)	7-40	39	30	33	33	19
GGT (U/L)	7-45	153	141	71	58	28
ALB (g/L)	40-55	43.9	37.9	48.2	48.0	38.7
T-bil (μmol/L)	0.0-21.0	14.0	9.7	18.9	16.6	10.9
D-bil (μmol/L)	0.0-8.0	5.5	4.9	4.1	5.2	4.4
PT (s)	11.0-14.3	13.7	14	12.8	12.9	12.7
Thyroid function						
TSH (mIU/L)	0.35-4.94	–	10.2122	11.0591	5.8207	4.3042
FT3 (pmol/L)	2.43-6.01	–	3.55	4.59	5.20	4.4800
FT4 (pmol/L)	9.01-19.05	–	11.26	12.02	12.28	13.3300
TPOAb (IU/mL)	0.00-5.61	–	2.09	4.48	3.22	0.4600
TGAb (IU/mL)	0.00-4.11	–	1.22	1.96	1.38	1.3500
Tumor marker						
AFP (ng/mL)	0.00-7.00	2.63	2.45	2.68	2.47	2.74
PIVKA (mAU/mL)	0.00-40.00	391.94	55.44	187.13	57.17	45.05
CA19-9(U/mL)	0.00-27.00	34.40	21.65	22.26	22.3	17.50

The low AFP level in this patient but high PIVKA level combined with the enhanced CT findings of HCC was considered first. The patient received the first TACE treatment in December 26, 2018: epirubicin 40 mg, fluorouracil 250 mg, lobaplatin 50 mg, lipiodol 15 mL, and gelatin sponge particles (350-560 μm). In January 1, 2019, oral sorafenib (400 mg b.i.d) was added and, in January 4, 2019, the immune checkpoint PD-1 inhibitor nivolumab (200 mg i.v biweekly (Q2W)) was added. In January 28, 2019, the patient received the second TACE treatment: epirubicin 40 mg, fluorouracil 250 mg, lobaplatin 50 mg, lipiodol 5 mL, and gelatin sponge particles (350-560 μm). The patient exhibited a good tolerance for the treatments. After 6 cycles of nivolumab (in March 27, 2019), the drug was replaced with toripalimab (240 mg, i.v Q3W) due to economic reasons. No immune-related adverse events occurred during the nivolumab treatment.

**Figure 3:**
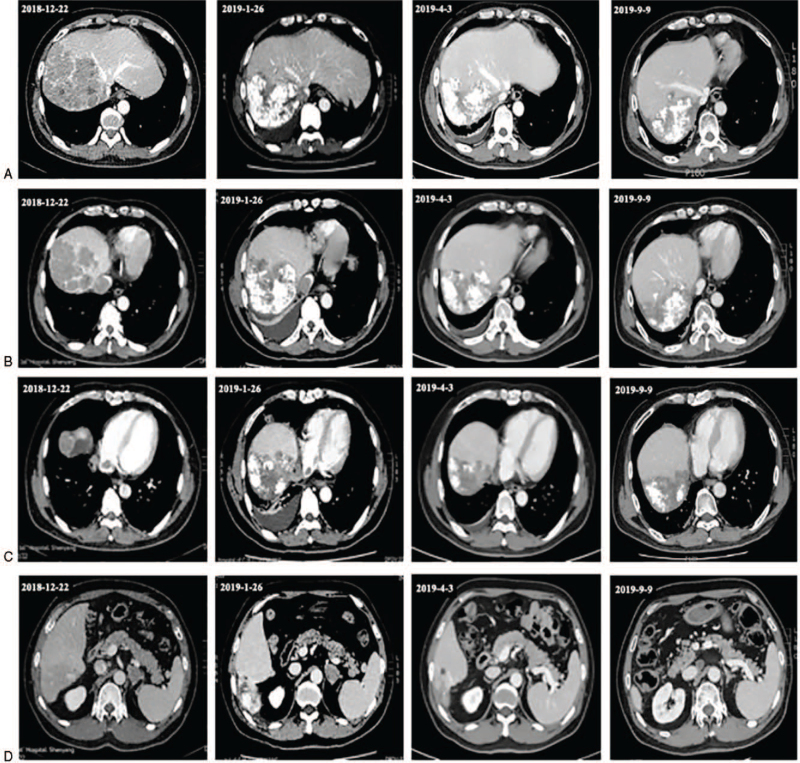
Tumor changes were observed by enhanced CT from December, 2018 to September, 2019. A: Lipiodol deposits are seen in the liver tumor, and the tumor has shrunk after treatment; B: The tumor thrombus of inferior vena cava shrank significantly; C: The right atrial tumor thrombus disappeared in April, 2019; D: Left adrenal gland metastasis disappeared in April 2019. CT = computed tomography.

In April 3, 2019, re-examination via enhanced abdominal CT revealed some obvious curative effects, such that the liver tumor size decreased, the IVC tumor thrombus exhibited significant shrinkage, and the RA tumor thrombus and the metastatic lesion of the left adrenal gland were significantly diminished. The laboratory test data revealed that the serum tumor markers AFP was 2.45 ng/mL, CA was 19-9 21.65 U/mL, and PIVKA was 55.44 mAU/mL (Table 1). We evaluated the tumor partial response (PR) based on the tumor assessment criteria mRECIST (Fig. 3 A-D). In April 8, 2019, the patient received a third TACE treatment: epirubicin 40 mg, fluorouracil 250 mg, lobaplatin 50 mg, lipiodol 5 mL, and embospheres (300-500 μm). It was also recommended that he continued the sorafenib and toripalimab treatment.

**Figure 4:**
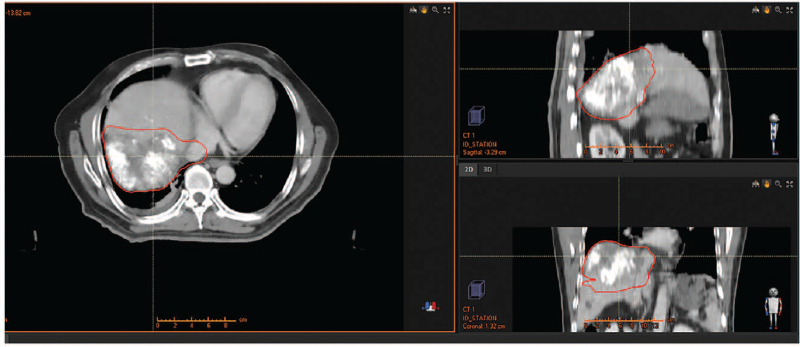
Region of the IGRT. IGRT = image-guided radiotherapy.

To further prolong the survival of the patient, we added radiotherapy in May 20, 2019: Image-guided radiotherapy (2 Gy × 28 cycle) of the liver tumor and IVC tumor thrombus (Fig. 4). Unexpectedly, after the 11th cycle of the immunotherapy and 12th cycle of the radiotherapy (June 20, 2019), the patient developed acute cholecystitis and underwent emergency laparoscopic cholecystectomy (the patient had a history of gallstones without obvious symptoms). The radiotherapy was withdrawn after the surgery, but the patient continued the sorafenib and toripalimab treatment.

**Figure 5:**
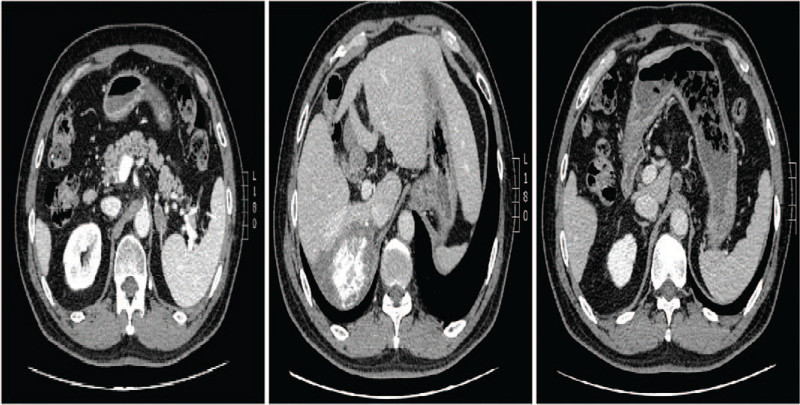
In April, 2020, enhanced CT showed that the left adrenal gland metastasis recurred, the lymph nodes of the hepatic hilar and retroperitoneal were enlarged. CT = computed tomography.

**Figure 6:**
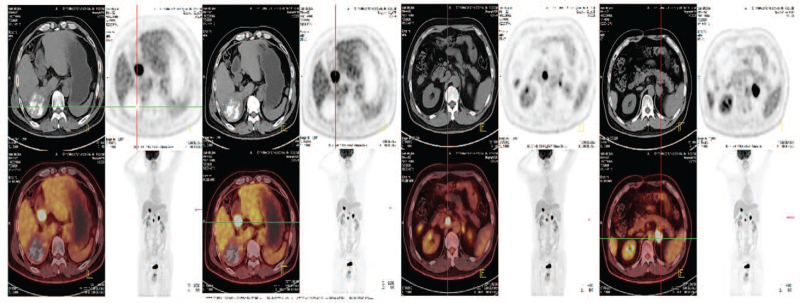
PET-CT showed the FDG of the left adrenal metastasis, hilar, and retroperitoneal enlargement lymph nodes were increased FDG, but there was no FDG intake in the liver and inferior vena cava area. FDG = fluorodeoxyglucose, PET-CT = positron emission tomography-CT.

In April 3, 2020, the patient's re-examination abdominal enhanced CT revealed that the left adrenal gland metastasis recurred and that the lymph nodes of the hepatic hilar and retroperitoneal were enlarged (Fig. 5). The laboratory test data revealed that the PIVKA was 187.13 mAU/mL, while AFP and CA 19-9 were normal (Table 1). The positron emission tomography-CT revealed that the fluorodeoxyglucose of the lymph node of hilar hepatic, retroperitoneal, and left adrenal gland were increased and that the largest standardized uptake value values were 18.1, 15.2, and 20.7, respectively. However, the mass in the right lobe and inferior vein were normal (Fig. 6). We evaluated the tumor progressive disease based on the tumor assessment criteria mRECIST.

**Figure 7:**
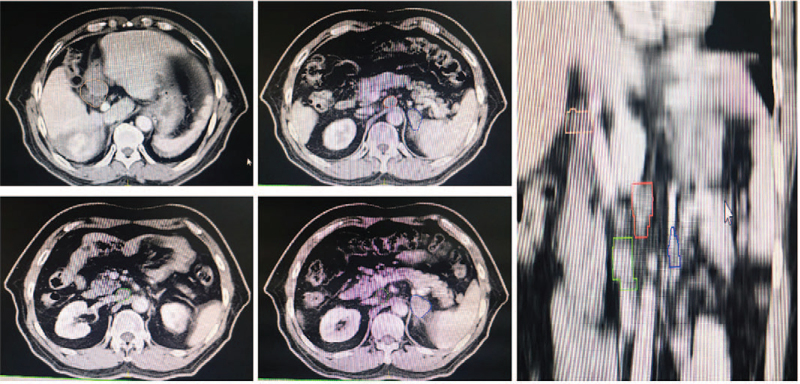
Region of the second IGRT (For the recurrent left adrenal gland metastasis, the enlarged hepatic hilar and retroperitoneal lymph nodes). IGRT = image-guided radiotherapy.

**Figure 8:**
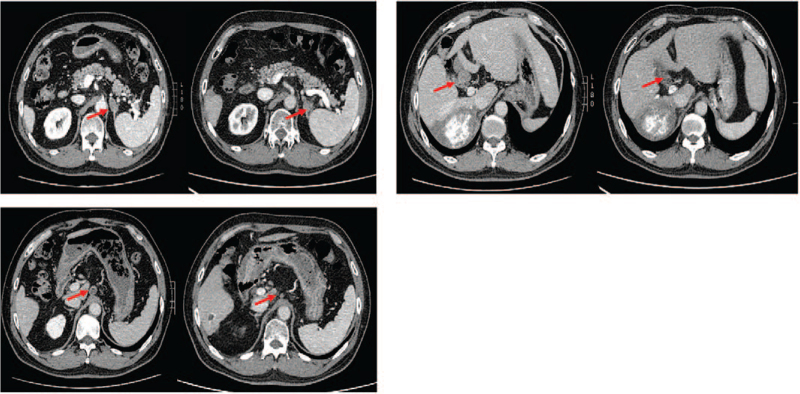
After the second IGRT, enhanced CT showed that the left adrenal gland metastasis, the lymph nodes of the hepatic hilar and retroperitoneal became obviously smaller. CT = computed tomography, IGRT = image-guided radiotherapy.

In order to prolong the patient's survival, we replaced sorafenib (400 mg b.i.d) with regorafenib (160 mg q.d) and choose RT in May 10, 2020: image-guided radiotherapy (2 Gy, 28 cycle of the lymph node of hilar hepatic and retroperitoneal and 2 Gy 30 cycle for the left adrenal gland [Fig. 7]). Fortunately, after the therapy, the patient had no adverse events, apart from a mild rash. In November 3, 2020, the patient re-examination abdominal enhanced CT revealed that the left adrenal gland metastasis recurred and that the lymph nodes of the hepatic hilar and retroperitoneal became obviously smaller (Fig. 8). In addition to this, the PIVKA reduced to 57.17 mAU/mL. We evaluated the tumor PR based on the tumor assessment criteria mRECIST. In July 27, 2021, the PIVKA reduced to 45.05 mAU/mL. Until now, we still follow-up the patient and the regorafenib and toripalimab treatment was maintained. The overall survival of this patient is now 34 months since the initial diagnosis of HCC.

## 3 Discussion

The prognosis of advanced HCC patients with high vascular invasion and distant metastasis is very poor, especially in advanced HCC patients with IVC or RA tumor thrombus, with a median survival of 1.9 to 5 months for untreated patients.^[11,12]^ Budd-Chiari syndrome is considered the most serious complication of HCC patients with IVC or RA tumor thrombus, with symptoms of acute right heart failure and pulmonary embolism.^[13,14]^ Extrahepatic metastasis is a predictor of poor prognosis in patients with advanced HCC (median survival is 8.9 months).^[15]^ Therefore, advanced HCC with IVC tumor thrombus, RA tumor thrombus, and distant metastasis is generally regarded as terminal. Tumor thrombus in the IVC and/or RA is usually classified into 3 types: type I = IVC tumor thrombus is below the diaphragm; type II = IVC tumor thrombus is above the diaphragm and under the atrium; type III = intracardiac. For type III tumor thrombus, surgery should be performed by liver and cardiothoracic surgeons under cardiopulmonary bypass.^[16]^

The patient in this case report was BCLC stage C with IVC tumor thrombus, RA tumor thrombus, and left adrenal gland metastasis. His RA tumor thrombus was type III, but was combined with left adrenal gland metastases. Currently, there is no general consensus on the proper management modality for this situation. According to the BCLC staging system, systemic therapy is recommended for BCLC stage C instead of curative treatment and sorafenib and lenvatinib treatment were recommended as the first-line treatment for this patient. Sorafenib is an oral multikinase inhibitor approved for the treatment of advanced HCC based on 2 randomized, placebo-controlled trials.^[5,7]^ However, sorafenib is only likely to delay tumor progression and the incidence of an objective response is very low (2%-3.3%).^[5,7]^ According to National Comprehensive Cancer Network guidelines, locoregional therapy (ablation, arterially directed therapies, and radiation therapy), clinical trials, systemic therapy, or supportive care is recommended for patients who are not suitable for surgery.^[17]^ Lenvatinib, the other first-line treatment for advanced HCC, is an inhibitor of vascular endothelial growth factor, fibroblast growth factor, platelet-derived growth factor, and other growth signaling targets. In a phase 3 open-label, multicenter noninferiority trial, the median overall survival associated with lenvtinib was 13.6 months, as compared to 12.3 months for sorafenib.^[6]^

A meta-analysis, including 27 studies from 1990 to 2017, revealed that sorafenib plus TACE may prolong the time to progression (TTP) and disease control rate (DCR) of unresectable HCC patients; however, the combination therapy may not significantly improve the overall survival (OS).^[18]^ In Gao's reports, it was found that sorafenib plus TACE can prolong the survival of HCC patients with IVC tumor thrombus.^[19]^ In addition, a meta-analysis demonstrated that the combination of sorafenib with TACE improved OS, objective response rate (ORR), TTP, and DCR when compared with TACE monotherapy alone.^[20]^

In recent years, immunotherapy has emerged as a major therapeutic modality in many solid tumors.^[21–30]^ Some clinical trials have demonstrated that some advanced HCC patient may benefit from PD-1 inhibitor.^[31,32]^ In the CheckMate-040 trial, the PD-1 inhibitor nivolumab resulted in a DCR of 55% and an ORR of 10% after 7.4 months follow-up in 28 patients with extrahepatic extension and vascular invasion.^[31]^ KEYNOTE-224 is a non-randomized, multicenter, open-label, phase 2 trial.^[32]^ In this trial, 104 eligible patients with advanced HCC who had previously been treated with sorafenib received pembrolizumab treatment and the results indicated that pembrolizumab might be a treatment option for these patients. A phase III randomized controlled trial of nivolumab vs sorafenib as first-line treatment in patients with advanced HCC (CheckMate 459, NCT02576509) and pembrolizumab as second-line treatment in Asian patients with advanced HCC (KEYNOTE-394, NCT03062358) are under way. In addition, there are studies showing that antiangiogenic combined anti-PD-1/PD-L1 therapy can change the tumor microenvironment from immunosuppressive to immunosupportive, thus stimulating the immune response and enhancing the efficacy of immunotherapy.^[33,34]^

In National Comprehensive Cancer Network guideline, RT was recommended as a curative and palliative treatment for patients with untestable or inoperable HCC.^[17,35–39]^ Some studies have demonstrated that TACE combined with external radiotherapy can improve local control and prolong survival of patients with advanced HCC better than TACE alone, sorafenib, or TACE combined with sorafenib.^[40–44]^ In Zeng's report, patients with IVC/RA tumor thrombus in RT group had longer survival than those in no-RT group.^[45]^ An observational study by Koo reported that advanced HCC patients with IVC tumor thrombus treated with TACE combined with 3-dimensional conformal radiotherapy had improved survival than those treated with TACE alone.^[46]^ Kim reported that TACE combined with RT in HCC patients with portal vein tumor thrombosis increased the median TTP and OS compared to TACE and sorafenib alone.^[47]^ Recently, Yoon reported that the progression-free survival rate was significantly higher in the TACE plus RT group than in the sorafenib group at 12 weeks (86.7% vs 34.3%), a significantly higher radiologic response rate than the sorafenib group at 24 weeks (33.3% vs 2.2%), a significantly longer median TTP (31.0 vs 11.7 weeks), and a significantly longer OS (55.0 vs 43.0 weeks).^[43]^ In a report by Yu et al,^[48]^ HCC patients who received a combination of nivolumab, TACE, sorafenib, and RT treatment had a significantly longer progression-free survival and OS than those who did not receive RT before or during nivolumab treatment. Recently, results of a meta-analysis, which compared between the option of surgery and RT for HCC patients with IVC/RA tumor thrombus, showed that the median OS in the surgery group was higher than that in the RT group (15.3 vs 11.7 months) and that the 1-year OS rate in the surgery group was higher than that in the RT group (62.4% vs 48.8%), but the 2-year OS rate was similar in the 2 groups (27.5% vs 26.9%).^[49]^ Recently, some studies reported that local RT could have effects on immune responses that induces the release of tumor-specific antigens, which result in anticancer immune responses that mediate abscopal effects, thereby increasing PD-L1 expression.^[50–57]^

In our case, the patient received 3 times TACE treatments and a combination of TKIs (sorafenib, regorafenib) and immunotherapy (nivolumab/toripalimab), followed by a second TACE treatment. After the TACE treatment, we combined image-guided RT to treat the liver tumor and IVC tumor thrombus. Unfortunately, the patient had cholecystitis during the radiotherapy, which led to the termination of radiotherapy. Immunotherapy can trigger immune-related adverse events, such as fatigue, diarrhea or colitis, dermatologic toxicities, endocrine toxicities (hypophysitis, hypothyroidism, hyperthyroidism, thyroiditis, and adrenal insufficiency), pneumonia, hepatic toxicities, pancreatic toxicities, renal toxicity, ocular toxicity, and neurologic syndromes, most of which are generally of low grade and easily manageable.^[58]^ In a report by Brown et al,^[59]^ HCC patients treated with immunotherapy exhibited a substantial increase in AST and/or ALT, but this did not result in cessation of therapy or cause death due to drug toxicity. During the treatment, related adverse events were slight and well-tolerated. Apart from a slight increase in gamma-glutamyl transpeptidase, the patient's ALT and AST were normal and the Child-Pugh class was A. During immunotherapy, the patient's TSH level was increased, but the levels of FT3, FT4, TPOAb, and TGAb were all in the normal range and the patient had no clinical symptoms of hypothyroidism. There were no serious adverse events or discontinuations due to adverse events.

Advanced HCC with adrenal glands metastatic are not common and the incidence is in the range of 8.8% to 16.9%.^[60,61]^ For the therapy of advanced HCC patients with adrenal glands metastases, surgical resection, ablation, and RT have been reported.^[62–64]^ RT is a good palliative therapy for advanced HCC patients with extrahepatic metastases.^[65–69]^ In a multi-institutional retrospective study conducted in Korea, for advanced HCC patients with adrenal metastasis treated with RT, the ORR was 38.3%, disease stability was 93.6%, and the adverse events were minimal.^[64]^ In our case, after the patient received 3 times TACE and 4 months sorafenib and PD-1 inhibitor treatments, the left adrenal metastases disappeared. However, a year later, in April 2020, we found that the metastases in the left adrenal gland, lymph node of hilar hepatic, and retroperitoneal recurred. After replaced sorafenib with regorafenib and combined with the RT treatment, metastases in the left adrenal gland, lymph node of hilar hepatic and retroperitoneal were reduced. Up to now, the patient is still alive.

## 4 Conclusion

The treatment for advanced HCC patients with portal vein and/or IVC and/or right atrium tumor thrombus should incorporate a personalized analysis of the pattern of tumor distribution. In our case, multimodal treatment showed an encouraging result and significantly prolonged the patient's survival. The liver tumor and IVC tumor thrombus were diminished and the RA tumor thrombus and metastatic lesion of the left adrenal gland disappeared. The patient achieved PR after 4 months of treatment. Our case suggests that this multimodal treatment for advanced HCC with IVC and RA tumor thrombus may be a suitable treatment option. However, further investigations in a larger sample of patients are required. Immunotherapy may be effective as adjuvant therapy or combination therapy for advanced HCC.

## Acknowledgments

Thanks to the patient for agree to publish his treatment process, and thanks to all the authors for their efforts in the treatment of this case.

## Author contributions

Jialin Zhang and Ailin Li conceived this study. Ning Sun performed data collection, conducted statistical analysis and prepared the original manuscript. Baifeng Li revised the original manuscript. Mutian Lv provided medical technical support. Chengshuo Zhang participated in data collection. All the authors have read and agreed to the final version of this manuscript.

**Data curation:** Ning Sun.

**Formal analysis:** Chengshuo Zhang, Ning Sun.

**Investigation:** Mutian Lv.

**Methodology:** Jialin Zhang, Ailin Li.

**Project administration:** Ning Sun.

**Resources:** Jialin Zhang, Mutian Lv.

**Supervision:** Jialin Zhang.

**Writing – original draft:** Ning Sun.

**Writing – review & editing:** Ning Sun, Baifeng Li.
